# A study on GHG emission assessment in agricultural areas in Sri Lanka: the case of Mahaweli H agricultural region

**DOI:** 10.1007/s11356-023-28488-8

**Published:** 2023-07-12

**Authors:** Hemali Rathnayake, Takeshi Mizunoya

**Affiliations:** 1 Doctoral Program in Sustainable Environmental Studies, Graduate School of Life and Environmental Sciences, University of Tsukuba, 1-1-1 Tennodai, Tsukuba, Ibaraki, 305-8572 Japan; 2 Faculty of Life and Environmental Sciences, University of Tsukuba, 1-1-1 Tennodai, Tsukuba, Ibaraki, 305-8572 Japan

**Keywords:** Agriculture, Livestock, Greenhouse gas emissions, Mitigation, Carbon sequestration, Net-zero emissions

## Abstract

Agricultural activities contribute 7% to Sri Lanka’s economy and account for 20% of the national greenhouse gas (GHG) emissions. The country aims to achieve zero net emissions by 2060. This study was aimed at assessing the present state of agricultural emissions and identifying mitigation strategies. The assessment involved estimating agricultural net GHG emissions from non-mechanical sources in the Mahaweli H region, Sri Lanka, in 2018 using the Intergovernmental Panel on Climate Change (IPCC 2019) guidelines. New indicators were developed to measure emissions for major crops and livestock and used to show the flow of carbon and nitrogen. The region’s agricultural emissions were estimated to be 162,318 t CO_2_eq y^−1^, of which 48% was from rice field methane (CH_4_) emissions, 32% from soil nitrogen oxide emissions, and 11% from livestock enteric CH_4_ emissions. Biomass carbon accumulation offset 16% of the total emissions. Rice crops exhibited the highest emission intensity of 4.77 t CO_2_eq ha^−1^ y^−1^, while coconut crop had the highest abatement potential of 15.58 t CO_2_eq ha^−1^ y^−1^. Approximately 1.86% of the carbon input to the agricultural system was released as carbon-containing GHG (CO_2_ and CH_4_), whereas 1.18% of the nitrogen input was released as nitrous oxide. The findings of this study suggest extensive adaptations of agricultural carbon sequestration strategies and increased nitrogen use efficiency to achieve GHG mitigation targets. The emission intensity indicators derived from this study can be used for regional agricultural land use planning to maintain designated levels of emissions and implement low-emission farms.

## Introduction

Agricultural activities have generated 12% of global net anthropogenic emissions (6.2 ± 1.4 GtCO_2_eq y^−1^) from 2007 to 2016, while contributing 40% of global methane (CH_4_) and 78% of nitrous oxide (N_2_O) emissions (IPCC 2020). To ensure food security for the growing population, global food production will need to increase by approximately 70% by 2050 (World Bank 2021). Thus, the tradeoff between agricultural production enhancement and achieving greenhouse gas (GHG) mitigation targets is challenging, yet obligatory. The United Nations Emission Gap Report (2021) highlighted that global GHG mitigation pledges will reduce projected emissions by only 7.5% in 2030, whereas reductions by 30 and 55% are required to keep global warming below 2 and 1.5 °C, respectively. Hence, all countries should explore their full potential to achieve net-zero pledges with near-term action plans (UNEP 2021).

The Intergovernmental Panel on Climate Change (IPCC 2020) reported that crops, livestock, and agroforestry-related activities have a total technical mitigation potential of 2.3–9.6 Gt CO_2_eq y^−1^ by 2050, revealing the feasibility of the agricultural sector to attain net zero. Thus, most countries should prioritize agricultural mitigation strategies to minimize emissions and maximize carbon sequestration by adopting sustainable land management practices. Agricultural GHG emissions stem from two major sources: non-mechanical sources driven by agricultural production-related biophysical processes and mechanical sources of machinery and equipment operations on farms (WRI 2014). The IPCC (2018) defines net-zero emissions as a complete balance between anthropogenic GHG emissions and their removal from the atmosphere. Therefore, balancing the agricultural GHG emissions in CO_2_eq with the potential carbon dioxide (CO_2_) removal via carbon (C) accumulation in plant biomass and soil can be defined as net zero for agricultural emissions. This study focuses on emissions from non-mechanical sources that release three main GHGs: methane (CH_4_), nitrous oxide (N_2_O), and carbon (IV) oxide (CO_2_).

Emission inventory and assessment are essential components of GHG mitigation policy planning and implementation. The IPCC (1996, 2006, and 2019) Guidelines for National Greenhouse Gas Inventories have provided the basic methodology for agricultural GHG estimation. The GHG assessment tools such as the Ex-Ante Carbon Balance tool, Source-selective and Emission-adjusted GHG calculator for cropland (SECTOR), Global Livestock Environmental Assessment Model (GLEAM), and Cool Farm tool have been developed using the IPCC and other simulation models and are available online to estimate farm or project level emissions.

### Agriculture and GHG emissions in Sri Lanka

Sri Lanka is a tropical island in South Asia, with a population of 22 million and land extending 66,610 km2, of which 40% is utilized for agriculture (Land Use and Policy Planning Department 2021). The country accounted for 0.08% of global GHG emissions, amounting to 33.7 Mt CO_2_ eq in 2018 (World Bank 2020). The United States Agency for International Development (USAID 2015) reported that the carbon intensity of Sri Lanka’s economy was approximately 1.5 times that of the world average. The country’s agriculture sector contributes approximately 7% to the GDP (Central Bank of Sri Lanka 2020), while contributing 20% to national GHG emissions (Ministry of Environment 2016). Rice is the major crop, grown in two monsoon rain seasons, accounting for over 12% of national land use. Field crops such as vegetables, legumes, yams, and perennial crops, including tea, rubber, coconut, fruits, and spices, are extensively cultivated as commercial crops. Dairy and poultry production dominates the livestock industry. According to the GHG emission assessment conducted by the Sri Lankan Ministry of Environment (2016), 52% of the country’s agricultural GHG emissions resulted from rice field CH_4_ emissions, 27% from livestock enteric CH_4_ emissions, and 16% from soil management. Sri Lanka has committed to reducing national GHG emissions by 14.5% from 2020 to 2030 and achieving net-zero emissions by 2060 (MOE 2021). Agricultural GHG mitigation is one of the most cost-effective and socially adaptive strategies to achieve these national targets.

As a tropical island in the Indian Ocean, which is highly vulnerable to extreme climate events, most climate-related studies on Sri Lanka’s agricultural sector have focused on vulnerability and adaptation planning. Less attention has been paid to identifying GHG mitigation strategies. Studies evaluating the carbon sequestration potential of perennial crops in Sri Lanka (Ranasinghe and Thimothias 2012; Mattsson et al. 2015; Wijeratne et al. 2014) are limited; however, Lokupitiya (2016) derived livestock CH_4_ emission factors, and the Food and Agriculture Organization for the United Nations (FAO 2017) assessed the GHG emissions and mitigation potential of the dairy sector. Many studies have highlighted the impact of rice-field emissions on global GHG accumulation by examining CH_4_ fluxes (Wassmann et al. 2009; Jiang et al. 2021; Rahman and Yamamoto 2020; Martínez-Eixarch et al. 2021; Gupta et al. 2021) and recommended mitigation options related to water management (Yamaguchi et al. 2016; Tapan et al. 2014; Prangbang et al. 2020). Soil nitrogen (N) dynamics and N_2_O fluxes (Wang et al. 2021; Kim et al. 2017; Chang et al. 2020), assessment of dairy sector emissions based on CH_4_ emission factors (Wolf et al. 2017; Kouazounde et al. 2015; Zhang et al. 2021), and total dairy production emissions (Bustamante et al. 2012; Vergé et al. 2007; Wilkes and Van Dijk 2018) have been investigated. The importance of C sequestration in biomass (Devi and Singh 2021; Drexler et al. 2021; Khan et al. 2021) and soil (Paustian et al. 2019; White 2022; Buck and Palumbo-Compton 2022; Vilakazi et al. 2022) to offset agricultural emissions is well documented. Farm-level emission performance (Syp and Osuch 2018; Feliciano et al. 2022; Stetter and Sauer 2022) and agricultural C and N cycles (Lal 2004; Udvardi et al. 2021; Fagodiya et al. 2017) have been evaluated in recent studies. Studies evaluating the impact of green investments on environmental performance in the agriculture, industry, and energy sectors discovered that a positive correlation exists between achieving carbon neutrality and promoting regional sustainability (Sharma et al. 2021; Lorente et al. 2023: Anu et al. 2023; Guo et al. 2021).

The evaluation of the net emission intensity of crops according to the IPCC guidelines is an area that has not been extensively covered by literature. While the IPCC guidelines are often used for GHG accounting projects, the potential use of these guidelines to identify strategy gaps needed for an urgent response toward agricultural emission mitigation remains unexamined. This study offers a new perspective to developing crop emission indicators by employing IPCC emission inventory guidelines in a local context and examining the C and N flow in an agricultural system.

In this study, we estimated the overall net GHG emissions from non-mechanical sources in the Mahaweli H agriculture region, Sri Lanka, for 2018, using the IPCC (2019) GHG inventory guidelines. Furthermore, by deriving the emission intensity indicators for major crops and livestock and demonstrating the associated C and N input-output flow, we attempted to identify strategic gaps and GHG mitigation pathways. The latest national agriculture GHG emission inventory for Sri Lanka (Ministry of Environment, Sri Lanka 2016) was prepared for the base year 2000 using the revised 1996 IPCC guidelines; however, it provides only national-level information. Therefore, updated and detailed information is required on a regional scale. Thus, the original contribution of this study is to provide necessary information on the present state of agricultural net GHG emissions and emission intensity of crop production and livestock on a regional scale using the latest IPCC guidelines, which would be a pioneering detailed regional agricultural GHG assessment in Sri Lanka.

**Fig. 1:**
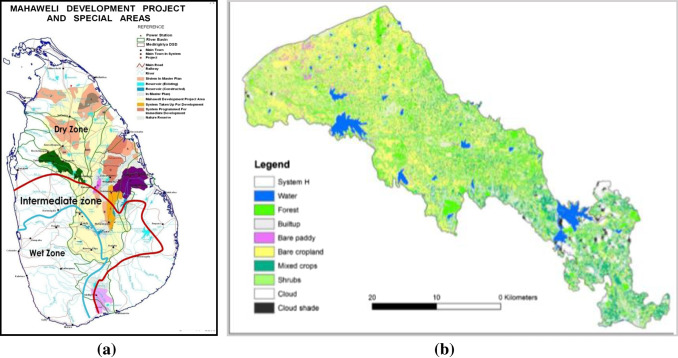
**a** Location of the study area. **b** Land use of the study area. Source of both figures: Mahaweli Authority of Sri Lanka 2019

The study area, which is the Mahaweli H agriculture region (areas colored green in Fig.1a and b), falls under the low country dry climate zone in the Anuradhapura district, North Central Province of Sri Lanka. The Anuradhapura district has the highest national rice production contribution (17%), with agricultural land allocation at 33% (Ministry of National Policies and Economic Affairs 2015). The Mahaweli H region is a predominantly agricultural area, resettled under the Mahaweli development program in 1980 with the provision of irrigation, which contributed 4.5% to the national rice production in 2018 (*Paddy Statistics* 2019). However, considering the applicability of the research results to a larger agricultural area and the feasibility of implementing regional or project level mitigation strategies in the Mahaweli administrative area, the region was selected for this study. Therefore, this study was aimed at assessing the present state of agricultural emissions and identifying mitigation strategies. The results can be used for top-down decision-making to incorporate GHG mitigation objectives into regional agricultural land use management planning. The ready availability and accessibility of such information will encourage farmers to voluntarily implement practices to reduce farm emissions.

## Methods

We employed IPCC 2019 agriculture GHG inventory methodology to the annual average crop production data of Mahaweli H agriculture region, Sri Lanka, in 2018 and estimated the total annual GHG emissions from crop and livestock production. Annual average emission intensity, GHG emissions per hectare of rice, other field crops, perennial crop cultivation, and livestock emissions per animal were assessed. Based on the total organic and inorganic fertilizer input, added crop residue, animal excretions, and gas releases, C and N flows in the system were demonstrated. Therefore, we attempted to identify better options for C and N management in the system and GHG mitigation pathways in the agricultural system. Detailed IPCC formulas used in GHG estimation are presented in the “GHG emission estimation methodology” section.

### Background of the study area

The study region, Mahaweli H agriculture system, included approximately 48,000 ha of agricultural land and provides settlements for 28,450 farming families (Mahaweli Authority 2019). The average annual precipitation was 1250 mm, with two monsoon rain seasons, and the average monthly temperature varied from 27 to 34 °C (Department of Meteorology 2022). Reddish brown earth and low humic gley soil were the predominant soil types in the study area (MASL 2019). Rice was the major crop, extending over 70% of annual cultivated lands with 145 cropping intensity in 2018 (MASL 2019). Additionally, other field crops (OFC), such as legumes and vegetables, and perennial crops, including fruits and coconuts, were cultivated commercially (Table 1).

**Table 1:** Cultivated land area and the production in 2018

Other field crops	Cultivation area (ha)	Production (t)	Average production (per season, t ha^−1^)
Paddy (in two seasons)	29,173	165,240	5.66
Other field crops			
Maize	854	3842	4.50
Soya beans	1387	3190	2.30
Millet	31	73	2.35
Pulses	87	128	1.47
Vegetables	1893	23,402	12.36
Potatoes and tubers	179	4154	23.21
Ground nut	102	203	1.99
Onion	90	898	9.98
Total other field crops	4623		
Perennial crops			
Banana	1655	22,088	13.35
Mango	704	2953	16.30
Coconut	1169	5802	4.96
Papaya & Other	259	3879	4.19
Total perennials	3787		

Source: Mahaweli Authority of Sri Lanka 2019

### Data collection

Cultivated land area and production-related data are based on the Mahaweli Socio-Economic Statistics of 2018 (Mahaweli Authority of Sri Lanka 2019) (Table 1). Crop fertilizer recommendations and crop budget data were obtained from the Department of Agriculture, Sri Lanka (2022). To obtain data on crop management practices, interviews and surveys were conducted with the Mahaweli System H Project Office, which was the administrative unit of the study area.

### GHG emission estimation methodology

In this study, the methodology for estimating GHG emissions described in the Agriculture, Forestry, and Other Land Use Section of the Refinement to the IPCC Guidelines for National GHG Inventories (IPCC 2019) was used to assess GHG emissions. The study area was a historical agricultural region, with no significant land use conversion within the past 20 years; therefore, it was considered the remaining cropland. Non-mechanical emissions of the four farming categories, (i) rice, (ii) other field crops, (iii) perennial crops, and (iv) livestock, which dominate the agricultural sector in the region, were included in the study. Accordingly, (i) CH_4_ emission from rice fields, (ii) direct and indirect N_2_O emissions from agricultural soil owing to nitrogen amendments, (iii) CO_2_ emissions from urea application, (iv) livestock-related emissions (including enteric CH_4_ emissions, CH_4_, and direct and indirect N_2_O emissions from manure management), and (v) C sequestration from biomass and soil were assessed to estimate the net GHG emissions of the agricultural system. Global warming potential of CO_2_, CH_4_, and N_2_O was adopted based on the values presented in the Fifth Assessment Report (AR5), 1, 28, and 265, respectively, and GHG emissions were estimated in terms of CO_2_ equivalents (CO_2_eq).

#### CH_4_ emissions from rice fields

The anaerobic decomposition of organic material in a flooded soil environment releases CH_4_ (IPCC 2006). Emissions primarily depend on water regime and organic carbon input, whereas soil type, weather, and field management practices are influential factors (Wassmann et al. 2009). The emissions were calculated using the IPCC (2019) Tier 1 approach (Eqs. 1, 2, and 3; Table 2). The IPCC (2019) default CH_4_ emission factor of 0.85 kg CH_4_ ha^−1^ day^−1^ was modified by scaling factors for the water regime during the cultivation period, water regime of the pre-cultivation period, and organic amendment. In the study area, irrigated water was provided during the growing season, allowing multiple drainage periods.

**Table 2:** Factors and values used in the estimation of CH_4_ emission from paddy cultivation

Factor		Value	Reference	Source
Annual harvested area (ha)	*A*	29,173	Includes two seasons	(MOSL 2018)
Cultivation period of rice (days)	*t*	112	Average in the study area	(MOSL 2018)
Baseline emission factor for continuously flooded fields without organic amendment (kg CH_4_ ha^−1^ d^−1^)	EF_C_	0.85	Value for South Asia	Table 5.1 (IPCC 2019)
Scaling factor to account for the differences in water regime during the cultivation period	SF_W_	0.55	Irrigated with multiple drainage periods	Table 5.12 (IPCC 2019)
Scaling factor to account for the differences in water regime in the pre-season before the cultivation period	SF_P_	1	Non-flooded pre-season < 180 days	Table 5.13 (IPCC 2019)
Application rate of organic amendment (t ha^−1^)	ROA_i_		Assume 80% of rice straw is returned	(MOSL 2018)
Crop residue		5.64		
Compost		10		
Conversion factor for organic amendment	CFOA_i_		Straw incorporated long (> 30 days) before cultivation	Table 5.14 (IPCC 2019)
Crop residue		0.19		
Compost		0.17

1 $$\mathrm{Annual}\ {\mathrm{CH}}_4\ \mathrm{emissions}=\mathrm{Adjusted}\ \mathrm{daily}\ \mathrm{emission}\ \mathrm{factor}\ \left({\mathrm{EF}}_i\right)\times \mathrm{Cultivation}\ \mathrm{period}\ (t)\times \mathrm{Area}\ \mathrm{cultivated}\ (A)$$

2 $$\mathrm{Adjusted}\ \mathrm{daily}\ \mathrm{emission}\ \mathrm{Factor}\ \left({\mathrm{EF}}_i\right)={\mathrm{EF}}_{\mathrm{C}}\times {\mathrm{SF}}_{\mathrm{W}}\times {\mathrm{SF}}_{\mathrm{P}}\times {\mathrm{SF}}_{\mathrm{O}}$$

3 $$\mathrm{Scaling}\ \mathrm{factor}\ \mathrm{for}\ \mathrm{organic}\ \mathrm{amendment}\left({\mathrm{SF}}_{\mathrm{O}}\right)={\left(1+{\sum}_{\mathrm{i}}{\mathrm{ROA}}_{\mathrm{i}}\times {\mathrm{CFOA}}_{\mathrm{i}}\right)}^{0.59}$$

#### Soil N_2_O emissions

Soil biochemical processes, including nitrification and denitrification, release N_2_O as a by-product (IPCC 2019). When a variety of organic and inorganic N fertilizers are applied to agricultural soils to increase crop productivity, soil N availability improves and intensifies the N_2_O release process (IPCC 2019). While some N is converted to N_2_O and directly released, another portion is lost through volatilization, leaching, and runoff, indirectly releasing N_2_O into the atmosphere (IPCC 2006). The amounts of soil N amendments from urea, crop residue, compost manure, and N released in soil organic matter (SOM) mineralization were used to estimate emissions. The recommended urea and compost application rates for the study region were used (Department of Agriculture Sri Lanka 2022), and it was assumed that 80% of the crop residue from the previous cultivation of rice and other field crops was incorporated into the same field. Accordingly, (i) direct and (ii) indirect N_2_O emissions from volatilization and (iii) indirect N_2_O emissions from leaching were calculated for major crops using the IPCC (2019) Tier 1 approach. (Eqs. 4, 5, and 6; Table 3).

4 $$\mathrm{Direct}\;{\mathrm N}_2\mathrm O\;\mathrm{emissions}=\left({\mathrm F}_{\mathrm{SN}}+{\mathrm F}_{\mathrm{CR}}+{\mathrm F}_{\mathrm{COMPST}}+{\mathrm F}_{\mathrm{SOM}}\right)\times{\mathrm{EF}}_1\times44/28$$

Indirect N_2_O emissions

5 $$\mathrm{From}\ \mathrm{volatilization}=\left[\left({\mathrm{F}}_{\mathrm{SN}}\times {\mathrm{F}\mathrm{rac}}_{\mathrm{VOL}}\right)+\left({\mathrm{F}}_{\mathrm{CR}}+{\mathrm{F}}_{\mathrm{COMPST}}\right)\times {\mathrm{F}\mathrm{rac}}_{\mathrm{VOL}}\right]\times {\mathrm{EF}}_2\times 44/28$$

6 $$\mathrm{From}\;\mathrm{leaching}=\left({\mathrm F}_{\mathrm{SN}}+{\mathrm F}_{\mathrm{CR}}+{\mathrm F}_{\mathrm{COMPST}}+{\mathrm F}_{\mathrm{SOM}}\right)\times{\mathrm F\mathrm{rac}}_{\mathrm{LEACH}}\times{\mathrm{EF}}_3\times44/28$$

**Table 3:** Factors used in estimating soil N_2_O emissions

Factors and reference			Value	Source
Direct N_2_O emission factor for N additions from synthetic fertilizers, organic amendments, and crop residues and N from SOM mineralization (kg N_2_O–N (kg N)^−1^)	Synthetic fertilizer	EF_1_	0.016	Table 11.1 (IPCC 2019)
	Other N input		0.006	
For flooded rice fields (kg N_2_O–N (kg N) ^−1^)	Single and multiple drainage		0.005	
Indirect N_2_O emission factor for N volatilization and redepositing (kg N_2_O–N (kg NH3–N + NO_X_–N volatilized) ^−1^)		EF_2_	0.014	Table 11.3 (IPCC 2019)
Indirect N_2_O emission factor for leaching/runoff (kg N_2_O–N (kg N leaching/runoff) ^−1^)		EF_3_	0.011	
Fraction for volatilization from synthetic fertilizer ((kg NH_3_–N + NOx–N) (kg N applied) ^−1^)		Frac_VOL_	0.15	
Fraction for volatilization from all organic N fertilizers applied ((kg NH_3_–N + NOx–N) (kg N applied or deposited)^–1^)			0.21	
Fraction for N losses by leaching/runoff in wet climates ((kg N (kg N addition) ^−1^)		Frac_LEACH_	0.24	

where, *F*_SN_ stands for annual amount of N in urea application (kg N y^−1^),*F*_CR_ annual amount of N in crop residue application (kg N y^−1^),*F*_COMPOST_ annual amount of N in compost fertilizer application (kg N y^−1^), and *F*_SOM_ annual amount of N released in soil organic matter mineralization (kg N y^−1^)

#### CO_2_ emissions from urea application

When urea (CO(NH_2_)_2_) is applied to soil or plants in the presence of moisture, it rapidly splits via hydrolysis and releases CO_2_ (Dias 2022). Hence, it is assumed that the CO_2_ fixed in the industrial production process of urea is released in agricultural fields (IPCC 2019). The recommended urea input data (Department of Agriculture Sri Lanka 2022) were used, and the emission factor (0.2) was equivalent to the carbon content of urea on an atomic weight basis (20% for CO(NH_2_)_2_; Eq. 7; IPCC 2006).

7 $$\mathrm{Annual}\ {\mathrm{CO}}_2\ \mathrm{emissions}=\mathrm{Annual}\ \mathrm{urea}\ \mathrm{amount}\ \mathrm{applied}\times \mathrm{EF}$$

#### Livestock emissions

Livestock emissions were calculated from three sources: (i) CH_4_ from enteric fermentation, (ii) CH_4_ from manure, and (iii) direct and indirect N_2_O from manure management. The study region included 4290 small-scale dairy farms, 129 goat farms that produced mutton, and 2590 poultry farms that produced eggs and chickens (MOSL 2019). Manure-related emissions were calculated by IPCC (2019) Tier 1 approach assuming 100% of manure was managed as solid storage.

CH_4_ from enteric fermentation: an enteric CH_4_ emission factor of 52 kg CH_4_ head^−1^ y^−1^, estimated by Lokupitiya 2016 for Sri Lankan dairy cattle in the dry zone using the Tier 2 approach of the IPCC (2006), was applied in this study. For goats, the IPCC default value of 5 kg CH_4_ head^−1^ y^−1^ was applied (EF_1;_ Eq. 8).

CH_4_ from manure management: manure-related CH_4_ emissions were estimated by multiplying the annual volatile solid (VS) excretion per head of animal species by the emission factor for the livestock category and the manure management system (Eq. 9).

N_2_O emissions from livestock manure management: direct emissions were estimated by multiplying the annual N excretion per head of animal species by the emission factor for the manure management system of the animal species, whereas indirect emissions were calculated by multiplying the fraction of N volatilizing or leaching by the emission factors. (Eqs. 10, 11, and 12; Table 4).

8 $$\mathrm{Enteric}\ {\mathrm{CH}}_4\mathrm{emissions}=\sum {\mathrm{EF}}_{1\left(\mathrm{i}\right)}\times \mathrm{No}\ {\mathrm{of}\ \mathrm{species}}_{\left(\mathrm{i}\right)}$$

9 $${\mathrm{CH}}_4\;\mathrm{emissions}\ \left(\mathrm{manure}\right)=\sum {\mathrm{EF}}_{2\left(\mathrm{i}\right)}\times \mathrm{No}\ {\mathrm{of}\ \mathrm{species}}_{\left(\mathrm{i}\right)}\times \mathrm{Annual}\ \mathrm{V}\ {\mathrm{excretion}\ \mathrm{rate}}_{\left(\mathrm{i}\right)}$$

10 $$\mathrm{Direct}\ {\mathrm{N}}_2\mathrm{O}\ \mathrm{emissions}\ \left(\mathrm{manure}\right)={\mathrm{EF}}_3\times \sum \left(\mathrm{No}\ {\mathrm{of}\ \mathrm{species}}_{\left(\mathrm{i}\right)}\times \mathrm{Annual}\ \mathrm{N}\ {\mathrm{excretion}\ \mathrm{rate}}_{\left(\mathrm{i}\right)}\right)\times\; 44/28$$

Indirect N_2_O emissions (manure)

11 $$\mathrm{From}\ \mathrm{volatilization}={\mathrm{EF}}_4\times \sum \left(\mathrm{No}\ {\mathrm{of}\ \mathrm{species}}_{\left(\mathrm{i}\right)}\times \mathrm{Annual}\ \mathrm{N}\ {\mathrm{excretion}\ \mathrm{rate}}_{\left(\mathrm{i}\right)}\times {\mathrm{Frac}}_{\mathrm{gasMS}\left(\mathrm{i}\right)}\right)\times 44/28$$

12 $$\mathrm{From}\ \mathrm{leaching}={\mathrm{EF}}_5\times \sum \left(\mathrm{No}\ {\mathrm{of}\ \mathrm{species}}_{\left(\mathrm{i}\right)}\times \mathrm{Annual}\ \mathrm{N}\ {\mathrm{excretion}\ \mathrm{rate}}_{\left(\mathrm{i}\right)}\right)\times {\mathrm{Frac}}_{\mathrm{leach}\ \mathrm{MS}}\times 44/28$$

**Table 4:** Factors used to estimate N_2_O emissions from livestock manure management

Factors			Value	Source
Volatile solid (VS) excretion per head of animal species (kg VS (1000 kg animal mass) ^−1^ d^−1^)	Dairy cattle		14.1	Table 10.13a (IPCC 2019)
	Goats		10.4	
	Poultry		14.9	
Enteric CH_4_ emission factor CH_4_ head^−1^ y^−1(^ kg CH_4_ head^−1^ y^−1^)	Dairy cattle		52	(Lokupitiya (2016)
	Goats		5	Table 10.14a (IPCC 2019)
CH_4_ emission factor for manure management system (g CH_4_ (kg VS) ^−1^)	Dairy cattle	EF_2_	4.4	Table 10.14a (IPCC 2019)
	Goats		4.4	
	Poultry		13.1	
Default N excretion rate (kg N (1000 kg animal mass) ^−1^ d^−1^)	Dairy cattle		0.65	Table 10.19 (IPCC 2019)
	Goats		0.34	
	Poultry		1.62	
Emission factor for direct N_2_O emissions the from manure management system		EF_3_	0.01	Table 10.21 (IPCC 2019)
Fraction of managed manure nitrogen for livestock category that volatilizes as NH_3_ and NO_x_ in the manure management	Dairy cattle	Frac_gasMS_	0.3	Table 10.22 (IPCC 2019)
	Goats		0.12	
	Poultry		0.4	
Fraction of managed manure nitrogen for livestock category that is leached from the manure management system	For all three species	Frac _leach MS_	0.2	Table 10.22 (IPCC 2019)
Emission factor for N_2_O emissions from atmospheric deposition of nitrogen on soils and water surfaces kg N_2_O-N (kg NH_3_-N + NO_x_-N volatilized) ^−1^		EF_4_	0.01	Table 11.3 (IPCC 2019)
Emission factor for N_2_O emissions from nitrogen leaching and runoff kg N_2_O-N (kg N leached and runoff) ^−1^		EF_5_	0.011	Table 11.3 (IPCC 2019)

#### Estimating C sequestration potential of agricultural systems

Biomass, dead organic matter, and soil represent the three C accumulation pools in agriculture. The IPCC (2019) methodology assumes that for annual crops, the increase in biomass stock is equal to the biomass loss from harvest and mortality in that same year; thus, no net C accumulation exists. Furthermore, the net C accumulation in below-ground biomass and dead organic matter in croplands was omitted in the IPCC Tier 1 approach. Thus, only perennial crops can sequester C via biomass accumulation. In the study area, the major commercial perennial crops were banana, coconut, mango, and papaya, covering over 94% of perennial croplands. Since the field-specific biomass accumulation information was limited, C accumulation rates for major perennial crops were obtained from previous studies (Ortiz-Ulloa et al. 2021; Ranasinghe and Thimothias 2012; Naik et al. 2019; IPCC 2006).

According to Ortiz-Ulloa et al. (2021), the residual content of banana crops after harvest is considered to be the net C sequestration of the system. The average residual to production ratio of 3.79, moisture content of 88.87%, and the carbon content of residue biomass of 40% (Ortiz-Ulloa et al. 2021) were applied to the average banana production of 13.35 t ha^−1^ y^−1^ in the study area; then, the C sequestration rate of the banana crop was assumed to be 2.25 t C ha^−1^ y^−1^. Ranasinghe and Thimothias (2012) estimated the C accumulation potential of monoculture coconut cultivation in Sri Lanka as 0.40 ± 0.19 t C ha^−1^ month^−1^. Boomiraj et al. (2020) derived a value of 4.89 t C ha^−1^ y^−1^ for the C accumulation potential of coconut plantations in Tamil Nadu, India. Consequently, this study used a value of 4.8 t C ha^−1^ y^−1^. Data on C accumulation for mango cultivation were obtained from Naik et al. (2019) and used in this study. Accordingly, biomass accumulation of 10-year-old mango cultivation, 1.05 t ha^−1^ y^−1^ (dry matter), was converted to 0.525 t C ha^−1^ y^−1^ by multiplying the C fraction by 0.5. It used the value 0.43 t C ha^−1^ y^−1^ for biomass C accumulation in papaya cultivation (IPCC 2006). The Rothamsted model developed by Coleman and Jenkinson (2014) for assessing soil C accumulation in non-waterlogged soil and the updated version for paddy soil by Shirato and Yokozawa (2005) were applied to calculate the soil organic carbon (SOC) accumulation rate.

## Results

### Estimating net GHG emissions

The emissions of the three GHGs from the main crop types, the total emissions in CO_2_eq, and the percentage contribution of each GHG to the total emissions were calculated (Table 5).

**Table 5:** Total GHG emissions from the main crop types in the agriculture system

Crop	Emissions (t y^−1^)				% of total (based on CO_2_eq)
	CH_4_	N_2_O	CO_2_	Total CO_2_eq	
Rice	3343.7	175.4	4599.6	144,713.9	74.4
OFC	-	29.6	650.9	8504.4	4.4
Perennial	-	32.9	812.4	9542.2	4.9
Livestock	901.0	24.8	-	31,794.1	16.3
Total	4244.7	262.8	6062.9	194,554.7	100.0
% based on CO_2_eq	61.1	35.8	3.1	100.0	-

The major crop, rice, occupying the largest share of the annual cultivated lands (77%, Table 1), accounted for a significant percentage (74.4%) of total annual emissions, while the livestock sector was the second largest emission source (16.3%). Furthermore, CH_4_ emitted by rice cropping and livestock farming was the highest-contributing GHG (61.1%) in the system, implying the need for improved C management strategies.

The carbon sequestration potential in the biomass and soil of each crop type was estimated on a per hectare basis, and the corresponding potential for CO_2_ abatement was determined (Table 6).

**Table 6:** Annual carbon sequestration and CO_2_ abatement potential of the study area

Crop	Biomass (t C ha^−1^ y^−1^)	Soil (t C ha^−1^ y^−1^)	Total (t C ha^−1^ y^−1^)	Abated CO_2_ (t ha^−1^ y^−1^)	Total land area (ha)	Total abated CO_2_ (t CO_2_ y^−1^)
Rice	-	0.0508	0.0508	0.186	15,590	2903.90
OFC	-	-0.4342	− 0.4342	− 1.592	4623	− 7360.12
Banana	2.25	0.0505	2.3005	8.435	1655	13,960.20
Coconut	4.8	0.0505	4.8505	17.785	1169	20,790.86
Mango	0.525	0.0505	0.5755	2.110	704	1485.56
Papaya	0.43	0.0505	0.4805	1.762	259	456.31
Total						32,236.71

Soil C sequestration rates, estimated by the Roth C carbon stock change model application (Coleman and Jenkinson 2014; Shirato and Yokozawa 2005), were 0.0508 t C ha^−1^ y^−1^ for rice fields and −0.4342 t C ha^−1^ y^−1^ for OFC. Slower anaerobic decomposition of organic matter in flooded rice fields helps to accumulate more carbon in the soil, resulting in a positive C sequestration rate while OFC fields showed an emission rate. For perennial crops, a single average value of 0.0505 t C ha^−1^ y^−1^ was obtained from the average of five commercial farms that reported soil quality in the study area.

Perennial crops showed greater CO_2_ abatement potential due to C accumulation in the biomass. With proper soil management, perennial crop fields can accumulate C in the soil. Coconut crops showed the highest abatement potential (17.785 CO_2_t ha^−1^ y^−1^), recommendable for the region to substantially offset the emissions. Since the net biomass C accumulation in rice and OFC was assumed to be zero, these crops could only abate emissions by soil C sequestration.

However, the annual soil C sequestration of the total system, estimated by multiplying the soil C sequestration rate per ha by cultivated lands, was negative, showing an emission of 1024 t C y^−1^, which is released through soil organic matter decomposition (Table 7). Agricultural soil management practices that improve soil biophysical structure and increase carbon sequestration should be adopted to limit soil carbon emissions and sustainable carbon management of the system, as an integral part of the net GHG mitigation strategy.

**Table 7:** Annual total soil C sequestration in the system

Crop	Total (t C ha^−1^ y^−1^)	Cultivation extent (ha)	Total (t C y^−1^)
Rice	0.0508	15,590	792
OFC	− 0.4342	4623	− 2007
Perennial	0.0505	3787	191
Total			− 1024

By deducting the total GHG abatements (32,236.71 t CO_2_ y^−1^) from the total emissions (194,554.7 t CO_2_eq y^−1^), the net emission of the agriculture system was estimated to be 162,318 t CO_2_eq y^−1^, representing 2.4% of the total agricultural emissions of the country in 2018. The system has the potential to offset 16% of the emissions by perennial biomass C accumulation, where it needs to be fully offset to attain net-zero emissions.

### Source-wise GHG emissions

The GHG emissions were estimated from the 6 sources presented in Table 8.

**Table 8:** Emissions by source

Emission source	Gas (t Ha^−1^ Yr^−1^)	CO_2_eq (t Ha^−1^ Yr^−1^)	% based on CO_2_ eq
CH_4_ from rice cultivation	3343.69	93,623.42	48.12
N_2_O from soil amendments (rice + other field crops + perennial)	238.02	63,074.22	32.42
CO_2_ from urea application (rice + other field crops + perennial)	6062.94	6,062.94	3.12
CH_4_ from livestock enteric fermentation	785.24	21,986.75	11.30
CH_4_ from livestock manure management	115.75	3241.10	1.67
N_2_O from livestock manure management	24.78	6566.26	3.38
Total		194,554.69	100.0

CH_4_ emissions from rice fields accounted for nearly half of the total non-mechanical emissions from the agricultural system. The larger land extent of rice fields (approximately 77% of the annual cultivated land in the region) resulted in a significant source-wise contribution. The daily CH_4_ emission factor for rice fields was estimated as 1.02 kg CH_4_ ha^−1^ day^−1^. Rice field CH_4_ emissions are mainly correlated with irrigation management, time, and amount of organic carbon amendments. The region has adapted to the irrigation water supply with multiple drainage periods, which reduces the emissions greatly compared to continuously flooded rice fields. Rice straw retention in the field might amend substantial organic carbon to the soil; however, because the amendment time is considered to be more than 1 month before the start of the growing season, it lowers the emission potential and increases soil C sequestration. Owing to the favorable climatic and physical conditions in the region, rice farming is more popular and therefore recommended for enhancement. Thus, the extent of the rice fields is to be maintained. Hence GHG mitigation strategies must focus on lowering the emissions per unit of cultivation land with improved straw management practices.

Soil N_2_O emission was the second largest contributor (32%) and was correlated with the amount of N amended in organic and inorganic forms. Direct and indirect soil N_2_O emission contributions were approximately 55 and 45% of the total amount; thus, lowering indirect emissions by limiting the N loss will significantly reduce total N_2_O emissions. Since organic fertilizer and crop residue are essential to maintain soil organic carbon level and favorable soil properties, reducing the synthetic fertilizer requirement by increasing the N use efficiency of the system through improved application methods and crop rotations is to be the main focus.

According to the IPCC (2019), the direct N_2_O emission factors for synthetic fertilizer and organic fertilizer were 0.016 and 0.006 kg N_2_O–N (kg N) ^−1^, respectively, and 0.005 kg N_2_O–N (kg N) ^−1^ for flooded fields. This implies that for upland crops, urea N causes more direct emissions than organic N input, and for rice fields, the impact is the same. Thus, reducing the urea N input will help to lower the direct emissions from OFC and perennial crops only.

The next largest emission contribution was made by livestock enteric emissions (11.3%), followed by the dairy sector. The adopted CH_4_ emission factor for dairy cattle was 52 CH_4_ head^−1^ y^−1^, a comparatively lower rate than that of the IPCC default value for the Indian subcontinent, 73 kg CH_4_ head^−1^ y^−1^. However, exploring the potential mitigation of enteric emissions via improved feed management practices is important for lowering the total emissions of the system. In the study region, CO_2_ from urea application and CH_4_ and N_2_O from livestock manure management represent lower contributions. Since the CO_2_ from urea application depends on the urea application rate, improved N management practices that reduce urea N requirement will also lower the CO_2_ release in the cropping field. Livestock manure emissions can be reduced by recovering energy and nutrient values.

### Estimating the emission intensity of main crops

The net emissions per hectare and net emissions per 1000 SLR (Sri Lankan Rupee) of farmer income gain were calculated (Table 9). Annual net emissions generated from 1 ha of crop cultivation were calculated by subtracting the CO_2_ abatement potential from the sum of CH_4_, N_2_O, and CO_2_ emissions in CO_2_eq. Rice exhibited the highest emission intensities in both net emissions per hectare and net emissions per 1000 SLR farmer income (4.77, 33.7 t CO_2_eq). All emission intensities of perennial crops (except for papaya) resulted in negative values, indicating their CO_2_ abatement potential. Coconut crop showed the highest abatement potential in both per hectare and per 1000 SLR farmer income. (15.58 t CO_2_eq, 51.94 t CO_2_eq). Since papaya cultivation requires a comparatively higher amount of urea fertilization, it resulted in higher N_2_O and CO_2_ emissions. Furthermore, papaya crop does not account for considerable biomass accumulation, thus resulting in an emission rate instead of abatement potential. This emphasizes that perennial crop selection for the region will also have a substantial impact on net agricultural emissions. According to this regional analysis, the emission intensities of rice and other crops are primarily varied with the C and N soil amendments. Thus, land productivity variations are influenced through annual crop residue input for rice and OFC, where urea and compost fertilizer inputs are determined at the regional scale. Therefore, considering the productivity difference range of ± 25%, possible emission variations for rice and OFC have been presented. For perennial crops, since the annul crop residue and litter input are determined by the farm management system, the impact of productivity variation for net emission was ignored. However, the factors of biomass C accumulation potential of crop species, N fertilizer requirements, C and N content of crop residue, and soil C accumulation potential of crop management practices are the key determinants of crop emission intensity.

**Table 9:** Emission intensity of major crops and net emissions per SLR 1000 of farmer income

Crop	Emissions (kg ha^−1^ y^−1^)				Sequestration (kg ha^−1^ y^−1^)	Net emissions per hectare (t ha^−1^ y^−1^)	*Net profit per hectare (SLR y^-1^)	Net emissions per SLR 1000 farmer income (kg CO_2_eq/SLR 1000)
	CH_4_	N_2_O	CO_2_	Total CO_2_eq	CO_2_	CO_2_eq	SLR (2018)	CO_2_eq
Rice	114.62	6.01	157.67	4961	186	4.77 ± 0.180	143,088	33.37
Maize		7.53	231.00	2228	− 1592	3.82 ± 0.026	218,950	17.45
Soya beans		4.78	91.67	1357	− 1592	2.95 ± 0.033	148,600	19.85
Millet		5.76	139.33	1665	− 1592	3.26 ± 0.033	400,090	8.14
Pulses		3.59	47.67	1000	− 1592	2.59 ± 0.013	314,475	8.24
Vegetables		8.44	165.00	2401	− 1592	3.99 ± 0.176	822,990	4.85
Sweet potato		7.40	88.00	2050	− 1592	3.64 ± 0.211	734,850	4.96
Manioc		6.24	165.00	1819	− 1592	3.41 ± 0.030	1,781,350	1.92
Ground nut		3.70	47.67	1027	− 1592	2.62 ± 0.020	195,825	13.38
Big onion		5.66	88.00	1589	− 1592	3.18 ± 0.96	1,820,350	1.75
Banana		10.41	310.93	3068	8435	− 5.370	1,450,831	− 3.70
Papaya		15.01	313.87	4293	1762	2.53	1,658,291	1.53
Coconut		7.65	176.00	2202	17785	− 15.58	300,000	− 51.94
Mango		4.54	24.20	1228	2110	− 0.88	1,012,101	− 0.87

*Net profit per ha of cultivation were adapted from the Department of Agriculture, Sri Lanka (2022)

### Livestock-related emissions

The annual emissions from livestock owing to enteric fermentation and manure management were estimated per head of animal species and added to the total population in the study area (Table 10). Accordingly, the total livestock-related emissions amounted to 31,794 t CO_2_eq y^−1^, while enteric CH_4_ emissions from dairy cattle were the major contributing source. The total production value of livestock, including milk, chicken, eggs, and mutton, in the region in 2018 was 3740 million SLR. Thus, the emission intensity of the total livestock production was 8.5 kg CO_2_ per 1000 SLR. This value is comparable to the emission intensity values of many cropping systems (Table 9; net emissions per SLR 1000 farmer income). Therefore, the integration of livestock will have a limited impact on the emission intensity of the regional economy; however, it will bring additional benefits if manure is properly managed to supply the N requirement for cropping systems. Since the estimation assumes that manure is totally managed as solid storage, the recovery of energy and N in manure will be an important pathway to circulate the resources among subsystems in the region. The region produced 5.8 million liters of milk in 2018; therefore, the emission intensity of milk production was calculated as 5.47 kg CO_2_eq per kg of fat and protein-corrected milk (FPCM), assuming a protein and fat content of 3.3% and 4%, respectively (FAO 2017). Table 10

**Table 10:** Emissions from livestock

Population category	No. of animal population	Emission per head of species (kg head^−1^ y^−1^)			Total annual emissions per head of species (kg head^−1^ y^−1^)	Total annual emissions (t CO_2_eq y^−1^)
		Emissions from enteric fermentation	Emissions from manure management			
		CH_4_	CH_4_	N_2_O	CO_2_eq	CO_2_eq
Dairy cattle	16,407	52	6.454	1.405	1868.94	30,663.76
Goats	4704	5	0.401	0.053	109.39	514.56
Poultry	112,036	-	0.071	0.013	5.50	615.78
Total			6.926			31,794.11

### Carbon and nitrogen flow in the system

The total C and N inputs to the system from each source are summarized in Table 11 which shows that compost fertilizer is the main C and N supplier in the agricultural system. This implies that the quantity and quality of compost will have a greater impact on C and N management. Biomass represents only a small portion of C accumulation in the total system; thus, extending perennial biomass via appropriate agricultural land use planning will be highly effective for carbon balancing in the system. Livestock manure adds a considerable N amount (1190 t y^−1^) that is similar to that (30%) added by urea into the system. Thus, the effective management of livestock manure as a nitrogen fertilizer to cropping fields can significantly reduce the synthetic N fertilizer requirement and thereby reduce associated emissions and indirect emissions of transportation, which are not included in these estimations.

**Table 11:** Amount of C and N input to the agricultural system

Input source	Total input (t y^−1^)	C input		N input	
		C % of the input source	C t y^−1^ added to the system	N % of the input source	N t y^−1^ added to the system
Compost	354,290	0.54	191,317	0.02	7086
Crop residue (dry matter)	277,000	0.4	110,800	*	2105
Urea	8268	0.2	1654	0.46	3803
Animal manure (dry matter)	3765	0.34	1280	**	1190
Biomass accumulation	-	-	9816	-	-
Total			314,866		14,184

*N input from crop residue was calculated using the values of Table 11.1A (IPCC 2019)

** input from animal manure has been calculated using N excretion rates of animal species (Table 10.19; IPCC 2019)

**Fig. 2:**
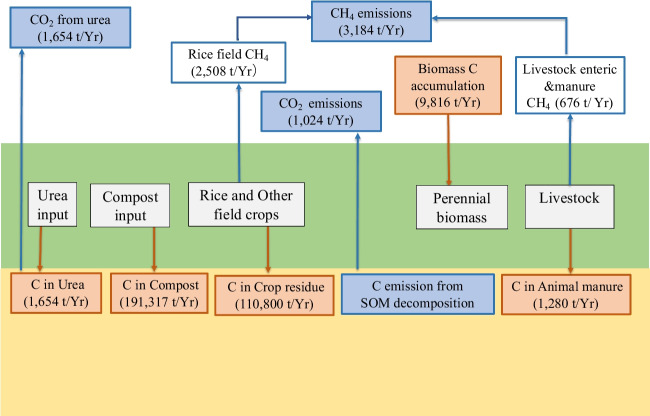
Carbon flow in the system

**Fig. 3:**
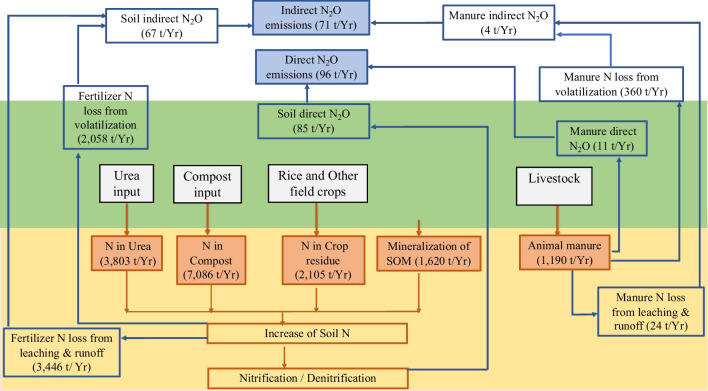
Nitrogen flow in the system

Carbon and N flows in the agricultural system are depicted in Figs. 2 and 3, respectively. Brown arrows represent the C and N inputs in the system, while blue arrows represent the emissions as GHGs. The numbers indicate the amount of C and N in the input sources and the GHG emissions in tons annually. The system intakes C mainly from compost and crop residue and emits 4244.7 t CH_4_ y^−1^, 6062.9 t CO_2_ y^−1^, and an additional 1024 t C y^−1^ via SOM decomposition, amounting to a total of 5862 t C y^−1^. The data show that 1.86% of total C input is released as C containing GHG emissions

The system takes up N from urea, compost, crop residue, animal manure, and SOM mineralization, approximately half of which is from compost. Estimations show that 5888 N t y^−1^ is lost from the system via volatilization, leaching, and runoff, which is 42% of the N loss, resulting in indirect N_2_O emissions. Thus, improving of the N utilization efficiency of agricultural systems is significant. The system emits a total of 262.8 t N_2_O y^−1^ and contains 167.2 N. Thus, 1.18% of the total N added to the system is released as direct and indirect N_2_O.

## Discussion

The GHG emission assessment showed that the Mahaweli H region’s agricultural sector requires significant modification to achieve net-zero emissions in the next few decades. Substantial CH_4_ emissions from extended rice fields have already limited the agricultural system’s GHG mitigation potential. The system potentially offsets only 16% of the emissions through biomass C accumulation, whereas it needs to be fully offset to achieve net-zero emissions. Refining the agricultural land use plan to accumulate more biomass C into the system, adopting soil S sequestration strategies, and increasing N use efficiency in the system are significant components of the regional agricultural GHG mitigation strategy.

According to Mahaweli Statistics (2018), the total agricultural production value of the study area was 15,906 million SLR, of which rice accounts for 30% of the total value while contributing to 74.4% of total emissions. Rice field CH_4_ emission was the highest-contributing source (48%) of the total emissions. The daily CH_4_ emission factor of rice fields was estimated as 1.02 kg CH_4_ ha^−1^ day^−1^; since the cultivation period was 112 days, the seasonal emission factor was 114 kg CH_4_ ha^−1^. IPCC (2019) has defined the value at 1.19 kg CH_4_ ha^−1^ day^−1^ (no flooding for less than 180 days prior to rice cultivation and continuously flooded during rice cultivation without organic amendment) as the world average, where South Asia has the lowest value of 0.85 compared to the values of 1.32 and 1.22 kg CH_4_ ha^−1^ day^−1^ for East Asia and Southeast Asia, respectively, implying that South Asia region should have a lower emission intensity compared to other regions. Saha et al. (2021) determined the seasonal emission factor for Bangladesh, using IPCC’s (2019) Tier 1 approach as 128–225 kg CH_4_ ha^−1^. Jiang et al. (2021) observed seasonal emissions in China for single cropping and ratooning early and late rice as 470, 330, and 220 kg CH_4_ ha^−1^, respectively, where intermittent irrigation and 100% crop residue are applied. In this study, the same emission rate was applied for two cultivation seasons in the year since no significant weather difference occurred throughout the year, with the field completely prepared again for the next season’s cultivation. The GHG emission intensity of rice in the study region was 4.77 t CO_2_ ha^−1^ for one season, and when applied to the seasonal average rice production, 5.66 t ha^−1^, the GHG emission intensity of rice production was 0.84 kg CO_2_eq kg^−1^ including 20 g of CH_4_ emissions. World Bank (2018) data showed that the emission intensity of rice varies from 0.6–2.4 kg CO_2_eq kg^−1^ in Asia to 0.7 kg CO_2_eq kg^−1^ in China and India.

To lower the rice field emission intensity per unit of cultivated land and per unit of production, feasible field management practices that minimize emissions and optimize yields must be explored. According to Weller et al. (2015), changing from a flooded system to an aerobic system causes a shift in the dominance of CH_4_ towards N_2_O emissions and consistently reduces crop yield, yet the high CH_4_ emissions of flooded cropping fields still override the enhanced N_2_O emissions in the aerobic upland system. Kim et al. (2017) demonstrated that, compared to non-planted soil, rice field soil acts as an N_2_O sink as N fertilizer levels increased. Gu et al. (2022) recommended the application of fermented manure instead of fresh straw and animal manure and the controlled release of synthetic fertilizer, thereby establishing a better soil microbial community to decrease CH_4_ emissions without influencing rice yield. Therefore, identification of the optimized organic and inorganic fertilizer mix and their application methods is imperative. The region has adapted to rice straw retention and is mixed with soil via mechanical tillage. Although straw input increases soil C levels and potential accumulation, it also intensifies the CH_4_ emissions. Thus, feasibility studies on using rice straw to produce fermented manure or biochar as field amendments are needed to select the best straw management options that lower the emissions and lead to retaining more C in the soil. Wassmann et al. (2009) also reported that flooded rice fields have a more favorable environment for forming conserved SOM and soil organic C levels. Han et al. (2016) showed that biochar amendment of rice fields could potentially reduce net emissions by 39.5%. Apart from the emission offset, the SOC level, which improves the soil properties, represents the sustainability of the ecosystem. Thus, the tradeoff of the three aspects of minimizing emissions, maximizing soil C sequestration, and optimizing yield is the main concern for emission mitigation in rice fields.

N_2_O from soil N amendments accounted for 32.4% of the total GHG emissions. The recommended urea application rates for each crop and N input from compost and crop residue resulted in distinct per hectare emission amounts. In Sri Lanka, adapting to fully organic fertilization is among the major concerns for improving crop production quality and limiting synthetic fertilizer imports; however, significant crop productivity reduction has been reported without synthetic fertilization (Torella 2022; Guzman 2022). In this analysis, based on IPCC calculation factors, net GHG mitigation benefits by fully adapting organic fertilization in the region were estimated using Eqs. 4, 5, and 6 (“Soil N2O emissions”) assuming that the total urea N input (3803 t y^−1^) was supplied by compost fertilizer. This resulted in a 5% reduction in N_2_O emissions, amounting to 1.8% of the total GHG mitigation in CO_2_eq. This proves that the transition to fully organic fertilization alone has a limited impact on reducing net emissions; thus, an optimum organic-inorganic fertilizer mix that mitigates emissions and increases production must be utilized.

Furthermore, our findings showed that the application of animal manure to cropping fields can reduce the urea N requirement by 30% in the agricultural system, yet the application method should lower the N loss. The N flow analysis showed that approximately 42% of the N input to the system was lost via volatilization, leaching, and runoff; therefore, increasing the N utilization efficiency of the systems must be considered. Anas et al. (2020) stated that N losses from agricultural fields due to excess fertilizer amendments, low plant populations, and poor application methods can reach up to 70% of the total available N and can be minimized to 15–30% by implementing improved agronomic practices. According to the IPCC (2019), approximately 1% of the N lost from the system is indirectly emitted as N_2_O. Estimates show that approximately 5888 t N y^−1^ is lost from the agricultural system; thus, halving that amount will save approximately 46 t N_2_O emissions, amounting to a 17.5% reduction in total N_2_O emissions and a 7.5% reduction in net emissions of the system in CO_2_eq annually. Mahmud et al. (2021) stated that measures such as the inclusion of nitrogen-fixing legumes in agricultural production, establishing artificial symbiosis or associative nitrogen fixation in non-legume plants, and efficient N management measures, such as the controlled release of N or drip N fertilization and applying 4Rs: right source, right rate, right time, and right placement, will improve the N utilization efficiency in cropping fields.

CH_4_ emissions from enteric fermentation accounted for 11.3% of the total emissions. The milk production emission intensity was estimated to be 5.47 kg CO_2_eq (kg FPCM) ^−1^. According to FAO (2017), the average emission intensity from milk production in Sri Lanka is 6.9 kg CO_2_eq (kg FPCM)^-1^, which includes emissions from feed production, transportation, and processing. A reduction potential of 10–29% in emission intensity and an increase of 15–45% in milk production by improving feed energy utilization and herd management (FAO 2017) exists. This study assumed that the manure was managed as solid storage, where the nutrient content or energy is not recovered, thus, contributing 5% of the total GHG emissions. The total annual emissions from enteric and manure per head of dairy cattle were 1.89 t CO_2_eq. Vergé et al. (2007) estimated that including CO_2_ emissions from farm field work-related energy consumption, GHG emissions per animal in the Canadian dairy sector were 4.55 t CO_2_eq. Manure biogas recovery and recycling for organic fertilizers are recommended so that synthetic fertilizer requirements and emissions related to transportation and operational activities can be minimized. However, additional emissions from feed management, grassland management, and farm operations will be important to realize the actual emissions from the livestock sector.

Total soil C sequestration in the agricultural system was estimated to be negative, indicating a lower C input than the decomposition rate, influenced by tillage practices. In the study area, crop residue application to cultivation fields is highly recommended for its nutrient value to increase yield and improve soil properties. However, more soil C sources such as biochar and green manure and management practices such as mulching, cover crops, and agroforestry must be promoted to maintain sustainable soil C sequestration. Because soil carbon supports soil nutrient sustainability, it further results in higher productivity enhancement. Kane (2015) recommended diverse crop rotations with cover crops and increased root biomass to maintain higher soil microbial biomass and C levels. Tillage practices (mechanical soil disturbance) also had a significant impact on SOC accumulation. A review by Haddaway et al. (2016) found that no-tillage practices compared to high tillage and intermediate tillage and intermediate tillage compared to high tillage can accumulate 2.09, 1.18, and 1.30 g C/kg, respectively. Huang et al. (2020) showed that the SOC accumulation potential of no-tillage with cover crop vs. conventional tillage with the cover crop was 0.089 versus 0.058 t C ha^−1^ y^−1^, respectively. However, SOC accumulation strongly depends on the physical properties of the soil, climatic factors, and C input level (FAO 2014). Therefore, field experiments are needed to determine the most favorable and feasible combination of practices.

According to the emission assessment results, C accumulation via perennial biomass is the only source that offsets emissions in the agricultural system where soil C sequestration shows a negative value. Therefore, refinement of the agricultural land use plan to extend the perennial crop carbon biomass is the most appropriate strategy for approaching net-zero emissions in the coming decades. The results revealed that the coconut crop, one of the most popular perennial crops in the region, has an abatement capacity of 15.58 CO_2_eq ha^−1^ y^−1^, indicating a greater opportunity. Promoting more perennial crops that are compatible with the regional soil and climatic conditions that bring more economic benefits to farmers and introducing a sustainable agroforestry farming system will be effective in long-term sustainability to maintain higher C biomass.

## Conclusion

This study estimated the total GHG emissions from non-mechanical sources of agricultural crop production and livestock activities in the Mahaweli H region, derived the emission intensity indicators for major crops and livestock, demonstrated the associated C and N flow, and attempted to identify strategic gaps to achieve GHG mitigation targets. The assessment showed that 74.4% of the total emissions stemmed from rice fields. Rice field CH_4_ emission was the highest-contributing source (48% of the total GHG in CO_2_eq). The agricultural system potentially offsets 16% of the emissions by perennial crop biomass. The results showed that rice had the highest emission intensity, whereas coconut crops had the highest abatement potential. Soil N_2_O emission was the second largest emission source (32% of total emissions), and N loss from the system was estimated to be 42% of the annual N amendments. Estimates showed that by shifting to fully organic fertilization, the total GHG emissions were lowered by only 1.8%. By properly managing animal manure N for cropping fields, the total urea N requirement of the system can be reduced by 30%. Soil C sequestration capacity of the agricultural system was estimated to be negative, with an emission rate of 1024 t C y^−1^ owing to SOM decomposition.

According to the assessment, the key points to be focused in regional agricultural GHG mitigation planning include (i) limiting net rice field emissions through productivity enhancement, adapting improved straw management options and soil amendments to lower emissions, and sequestrating more C to the soil; (ii) increasing the N use efficiency of the system by adapting manure utilization, improved application methods, and crop rotations, thereby reducing synthetic N requirements; (iii) adapting soil C sequestration management practices; and (iv) increasing perennial crop biomass and promoting agroforestry by integrated agricultural land use planning. The emission intensity indicators developed in this study can be readily employed in decision-making at the farm and regional levels to evaluate the present and potential emission performances in irrigated dry zones and are adjustable for other areas.

However, because the assessment was limited to non-mechanical emission sources, mechanical emissions from farm energy consumption and livestock feed management were not included. In addition, the biological factors of C and N exchange through respiration, harvest removal, and N fixation, which were not included in the adopted methodology, have been omitted. Relying on most IPCC emission factors that have not been verified for the region will have an impact on realizing accurate net emissions. Thus, the results are better suited for guiding regional agricultural land-use management and planning to incorporate GHG mitigation objectives. Further studies on deriving national, regional, and climate zone-specific emission factors for the country and inclusion of emissions from mechanical sources will help to develop precise emission and sequestration indicators. Compatibility analysis of mitigation potential and farmer adaptability for various low-emission agronomic practices and C sequestration strategies, including agroforestry, green manure application, biochar amendments, and perennialization, would be important in realizing a specific net-zero emission transition plan for the agriculture sector in Sri Lanka.

## Data Availability

All collected data and materials are reported in the manuscript.

## References

[CR1] Anas M, Liao F, Verma KK, Sarwar MA, Mahmood A, Chen ZL, Li Q, Zeng XP, Liu Y, Li YR (2020). Fate of nitrogen in agriculture and environment: agronomic, eco-physiological and molecular approaches to improve nitrogen use efficiency. Biol Res.

[CR2] Anu SAK, Singh AK, Raza SA, Nakonieczny J, Shahzad U (2023). Role of financial inclusion, green innovation, and energy efficiency for environmental performance? Evidence from developed and emerging economies in the lens of sustainable development. Struct Change Econ Dyn.

[CR3] Boomiraj K, Jagadeeswaran R, Karthik S, Poornima R, Jothimani S, Sudhagar RJ (2020). Assessing the carbon sequestration potential of coconut plantation in Vellore district of Tamil Nadu, India.

[CR4] Buck HJ, Palumbo-Compton A (2022). Soil carbon sequestration as a climate strategy: what do farmers think?. Biogeochemistry.

[CR5] Bustamante MMC, Nobre CA, Smeraldi R, Aguiar APD, Barioni LG, Ferreira LG, Longo K, May P, Pinto AS, Ometto JPHB (2012). Estimating greenhouse gas emissions from cattle raising in Brazil. Clim Change.

[CR6] Cai X, Bastiaans W (2018) Water productivity assessment for improved irrigation performance and water security in the Asia-Pacific Region: Sri Lanka technical report

[CR7] Central Bank of Sri Lanka (2020). Annual report. https://www.cbsl.gov.lk/en/publications/economic-and-financial-reports/annual-reports/annual-report-2020

[CR8] Chang N, Zhai Z, Li H, Wang L, Deng J (2020) Impacts of nitrogen management and organic matter application on nitrous oxide emissions and soil organic carbon from spring maize fields in the North China Plain. Soil Till Res 196. 10.1016/j.still.2019.104441

[CR9] Coleman K, Jenkinson DS (1987). A model for the turnover of carbon in soil: model description and users guide. Rothamsted Res Harpenden Herts.

[CR10] Coleman K, Jenkinson DS (2014). RothC—a model for the turnover of carbon in soils. Model Description and User Guide.

[CR11] Deininger DU (2022) Greening the rice we eat, World Bank Blogs*.* https://blogs.worldbank.org/eastasiapacific/greening-rice-we-eat. Accessed 15 Mar 2022

[CR12] Department of Agriculture (2022) Sri Lanka. Crop technology. https://doa.gov.lk/crop-production/

[CR13] Department of Census and Statistics Sri Lanka (2020) Paddy statistics 2019. https://statistics.gov.lk/agriculture

[CR14] Department of Metrology (2022) Sri Lanka. Weather and climate data. http://www.meteo.gov.lk/index.php?option=com_content&view=article&id=100&catid=21&lang=en&Itemid=321

[CR15] Devi AS, Singh KS (2021). Carbon storage and sequestration potential in aboveground biomass of bamboos in North East India. Sci Rep.

[CR16] Dias DR (2022) Understanding the chemical reaction of urea in the soil, Agronomy e Updates March 10th Issue 896. https://eupdate.agronomy.ksu.edu/issue_new/k-state-agronomy-eupdate-issue-896-thu-mar-10-2022

[CR17] Drexler S, Gensior A, Don A (2021). Carbon sequestration in hedgerow biomass and soil in the temperate climate zone. Reg Environ Change.

[CR18] Fagodiya RK, Pathak H, Kumar A, Bhatia A, Jain N (2017). Global temperature change potential of nitrogen use in agriculture: a 50-year assessment. Sci Rep.

[CR19] Feliciano D, Recha J, Ambaw G, MacSween K, Solomon D, Wollenberg E (2022). Assessment of agricultural emissions, climate change mitigation and adaptation practices in Ethiopia. Clim Policy.

[CR20] Food and Agriculture Organization of the United Nations (2014) Global Soil Organic Carbon Map, i8891en.pdf (fao.org)

[CR21] Food and Agriculture Organization for the United Nations (2017) Options for low emission development of Sri Lanka Dairy Sector. https://www.fao.org/3/i7673e/i7673e.pdf

[CR22] Greenhouse gas protocol (2020). Global warming potential values. https://www.ghgprotocol.org/sites/default/files/ghgp/Global-Warming-Potential-Values%20%28Feb%2016%202016%29_1.pdf

[CR23] Gu X, Weng S, Li Y, Zhou X (2022). Effects of water and fertilizer management practices on methane emissions from paddy soils: synthesis and perspective. Int J Environ Res Public Health.

[CR24] Guo J, Zhou Y, Ali S, Shahzad U, Cui L (2021). Exploring the role of green innovation and investment in energy for environmental quality: an empirical appraisal from provincial data of China. J Environ Manage.

[CR25] Gupta K, Kumar R, Baruah KK, Hazarika S, Karmakar S, Bordoloi N (2021). Greenhouse gas emission from rice fields: a review from Indian context. Environ Sci Pollut Res Int.

[CR26] Guzman CD (2022) The crisis in Sri Lanka rekindles debate over organic farming. How Organic Farming Worsened Sri Lanka’s Economic and Political Crisis | Time

[CR27] Haddaway NR, Hedlund K, Jackson LE, Kätterer T, Lugato E, Thomsen IK, Jørgensen HB, Isberg PE (2016) How does tillage intensity affect soil organic carbon? A systematic review protocol. Environ Evid 5. 10.1186/s13750-016-0052-0

[CR28] Han X, Sun X, Wang C, Wu M, Dong D, Zhong T, Thies JE, Wu W (2016). Mitigating methane emission from paddy soil with rice-straw biochar amendment under projected climate change. Sci Rep.

[CR29] Hitihamu S, Epasingha S (2015) Socio economic condition of dairy industry in Mahaweli H area. Rep. Sri Lanka: Hector Kobbekaduwa agrarian research and training institute. http://www.harti.gov.lk/images/download/reasearch_report/new1/184.pdf, p 163

[CR30] Huang, G, Heokstra AY, Krol MS, Galindo A, Jägermeyr J, Yu C, Wang R (2020) Water-saving agriculture can deliver deep water cuts for China. Resour Conserv Recycl 154:104578. 10.1016/j.resconrec.2019.104578

[CR31] Intergovernmental Panel on Climate Change (IPCC) (2006) IPCC guidelines for national greenhouse gas inventories, vol 2006*.* https://www.ipcc-nggip.iges.or.jp/public/2006gl/vol4.html

[CR32] IPCC (2018) Summary for policymakers. In: Glob Warming of 1°C. https://www.ipcc.ch/site/assets/uploads/sites/2/2022/06/SPM_version_report_LR.pdf

[CR33] IPCC (2019) Refinement to the 2006 guidelines for national greenhouse gas inventories. https://www.ipcc-nggip.iges.or.jp/public/2006gl/vol4.html

[CR34] IPCC (2020) Climate change and land: summary for policy makers. https://www.ipcc.ch/site/assets/uploads/sites/4/2020/02/SPM_Updated-Jan20.pdf

[CR35] Jiang Y, Zhang H, He J, Huan P (2021). Study on methane emission factor of paddy fields in Hubei Province. IOP Conf Ser Earth Environ Sci.

[CR36] Kane D (2015) Carbon sequestration potential on agricultural lands: a review of current science and available practices. https://sustainableagriculture.net/wp-content/uploads/2015/12/Soil_C_review_Kane_Dec_4-final-v4.pdf

[CR37] Khan I, Hayat U, Mujahid A, Majid A, Chaudhary A, Badashan MT, Huang J (2021). Evaluation of growing stock, biomass and soil carbons and their association with a diameter: a case study from a planted chir pine (pinus roxburghii) forest. Appl Ecol Environ Res.

[CR38] Kim SU, Choi EJ, Jeong HC, Lee JS, Lee HH, Park HJ, Hong CO (2017). Nitrogen dynamics in soil amended with different rate of nitrogen fertilizer. Korean J Soil Sci Fert.

[CR39] Kouazounde JB, Gbenou JD, Babatounde S, Srivastava N, Eggleston SH, Antwi C, Baah J, McAllister TA (2015). Development of methane emission factors for enteric fermentation in cattle from Benin using IPCC Tier 2 methodology. Animal.

[CR40] Lal R (2004). Agricultural activities and the global carbon cycle. Nutr Cycl Agroecosystems.

[CR41] Land use policy planning department Sri Lanka (2021). National Land Use*.* https://luppd.gov.lk/images/content_image/downloads/pdf/national_land_use_policy.pdf*.* Policy Press

[CR42] Lokupitiya E (2016). Country-specific emission factors for methane emission from enteric fermentation: a case study from a non-annex 1 country. J Natn Sci Found Sri Lanka.

[CR43] Lorente DB, Mohammed KS, Cifuentes-Faura J, Shahzad U (2023). Dynamic connectedness among climate change index, green financial assets and renewable energy markets: novel evidence from sustainable development perspective. Renew Energy.

[CR44] Mahaweli Authority of Sri Lanka (2018) 2018 Socio Economic Statistics.

[CR45] Mahaweli Authority of Sri Lanka (2019) 2018 Socio economic statistics*.* http://mahaweli.gov.lk/PDF/Statistical%20Book%20-%202018%20Final.pdf

[CR46] Mahmud K, Panday D, Mergoum A, Missaoui A (2021). Nitrogen losses and potential mitigation strategies for a sustainable agroecosystem. Sustainability.

[CR47] Martínez-Eixarch M, Alcaraz C, Viñas M, Noguerol J, Aranda X, Prenafeta-Boldú FX, Català-Forner M, Fennessy MS, Ibáñez C (2021). The main drivers of methane emissions differ in the growing and flooded fallow seasons in Mediterranean rice fields. Plant Soil.

[CR48] Mattsson E, Ostwald M, Nissanka SP, Pushpakumara DKNG (2015). Quantification of carbon stock and tree diversity of homegardens in a dry zone area of Moneragala district, Sri Lanka. Agroforest Syst.

[CR49] Ministry of National Policies and Economic Affairs (2015) Economic census. Agricultural activities. https://statistics.gov.lk

[CR50] Ministry of the Environment (2016) Sri Lanka. Second national communication on climate change. https://unfccc.int/sites/default/files/resource/lkanc2_0.pdf

[CR51] Ministry of the Environment Sri Lanka (2021) Updated nationally determined contribution*.* http://www.climatechange.lk/CCS2021/UpdatedNDCsSriLanka2021.pdf

[CR52] Naik SK, Sarkar PK, Das B, Singh AK, Bhatt BP (2019). Biomass production and carbon stocks estimate in mango orchards of hot and sub-humid climate in eastern region, India. Carbon Manag.

[CR53] Ortiz-Ulloa JA, Abril-González MF, Pelaez-Samaniego MR, Zalamea-Piedra TS (2021). Biomass yield and carbon abatement potential of banana crops (*Musa* spp.) in Ecuador. Environ Sci Pollut Res Int.

[CR54] Paustian K, Larson E, Kent J, Marx E, Swan A (2019). Soil C sequestration as a biological negative emission strategy. Front Clim.

[CR55] Prangbang P, Yagi K, Aunario JKS, Sander BO, Wassmann R, Jäkel T, Buddaboon C, Chidthaisong A, Towprayoon S (2020) Climate-based suitability assessment for methane mitigation by water saving technology in paddy fields of the central plain of Thailand. Front Sustain Food Syst 4. 10.3389/fsufs.2020.575823

[CR56] Rahman MM, Yamamoto A, Meena RS (2020). Methane cycling in paddy field: a global warming issue. Agrometeorology.

[CR57] Ranasinghe CS, Thimothias KSH (2012). Estimation of carbon sequestration potential in coconut plantations under different agro-ecological regions and land suitability classes. J Natn Sci Foundation Sri Lanka.

[CR58] Saha MK, Mia S, Biswas AAA, Sattar MA, Dijkstra FA (2021) Methane emission from different rice cultivation systems in Bangladesh: a model-based approach. Res Square. 10.21203/rs.3.rs-552062/v1

[CR59] Sharma GD, Shah MI, Shahzad U, Jain M, Chopra R (2021). Exploring the nexus between agriculture and greenhouse gas emissions in BIMSTEC region: the role of renewable energy and human capital as moderators. J Environ Manage.

[CR60] Shirato Y, Yokozawa M (2005). Applying the Rothamsted carbon model for long-term experiments on Japanese paddy soils and modifying it by simple tuning of the decomposition rate. Soil Sci Plant Nutr.

[CR61] Stetter C, Sauer J (2022). Greenhouse gas emissions and eco-performance at farm level: a parametric approach. Environ Resour Econ.

[CR62] Syp A, Osuch D (2018). Assessing greenhouse gas emissions from conventional farms based on the farm accountancy data network. Pol J Environ Stud.

[CR63] Tapan KA, Linquist B, Searchinger T, Wassmann R, Xiaoyuan Y (2014). Wetting and Drying: Reducing Greenhouse Gas Emissions and Saving Water from Rice Production.

[CR64] The World Bank (2021) Climate smart agriculture. https://www.worldbank.org/en/topic/climate-smart-agriculture

[CR65] Torella K (2022) A shift to better farming practices is possible, but Sri Lanka’s abrupt switch to organics offers a bitter lesson in how to change food systems in a sustainable way. Sri Lanka’s organic farming disaster, explained - Vox

[CR66] Udvardi M, Below FE, Castellano MJ, Eagle AJ, Giller KE, Ladha JK, Liu X, Maaz TM, Nova-Franco B, Raghuram N, Robertson GP, Roy S, Saha M, Schmidt S, Tegeder M, York LM, Peters JW (2021) A research road map for responsible use of agricultural nitrogen. Front Sustain Food Syst 5. 10.3389/fsufs.2021.660155

[CR67] United Nations Environment Programme (2021) The heat is on: emission gap report 2021*.* https://www.unep.org/resources/emissions-gap-report-2021

[CR68] United States Agency for International Development (2015) Greenhouse gas emission in Sri Lanka. https://pdf.usaid.gov/pdf_docs/pa00msrq.pdf

[CR69] Vergé XPC, Dyer JA, Desjardins RL, Worth D (2007). Greenhouse gas emissions from the Canadian dairy industry in 2001. Agric Syst.

[CR70] Vilakazi BS, Zengeni R, Mafongoya P (2022). Tillage and urea fertilizer application impacts on soil C fractions and sequestration. Agronomy.

[CR71] Wang C, Amon B, Schulz K, Mehdi B (2021). Factors that influence nitrous oxide emissions from agricultural soils as well as their representation in simulation models: a review. Agronomy.

[CR72] Wassmann R, Hosen Y, Sumfleth K (2009). Agriculture and climate change: reducing methane emissions from irrigated rice.

[CR73] Weller S, Kraus D, Ayag KRP, Wassmann R, Alberto MCR, Butterbach-Bahl K, Kiese R (2015). Methane and nitrous oxide emissions from rice and maize production in diversified rice cropping systems. Nutr Cycl Agroecosyst.

[CR74] White RE (2022). The role of soil carbon sequestration as a climate change mitigation strategy: an Australian Case Study. Soil Syst.

[CR75] Wijeratne T, De Costa J, Wijeratne M (2014) Carbon sequestration potential of tea plantations in Sri Lanka. Conference: 228th experiment and extension forum at Colombo, 228 (Conference paper) Available https://www.researchgate.net/publication/261229546_Carbon_Sequestration_Potential_of_TeaPlantations_in_Sri_Lanka

[CR76] Wilkes A, Van Dijk S (2018) Tier 2 inventory approaches in the livestock sector: a collection of agricultural greenhouse gas inventory practices. CGIAR Research Program on Climate Change, Agriculture and Food Security, Wageningen, The Netherlands (CCAFS) https://hdl.handle.net/10568/109610.

[CR77] Wolf J, Asrar GR, West TO (2017). Revised methane emissions factors and spatially distributed annual carbon fluxes for global livestock. Carbon Balance Manag.

[CR78] Word Resource Institute (2014) GHG protocol agriculture guidance. https://ghgprotocol.org/sites/default/files/standards/GHG%20Protocol%20Agricultural%20Guidance%20%28April%2026%29_0.pdf

[CR79] World B (2020) The total GHG emissions Sri Lanka. https://data.worldbank.org/indicator/EN.ATM.GHGT.KT.CE?locations=LK

[CR80] Yamaguchi T, Tuan LM, Minamikawa K, Yokoyama S (2016). Alternate wetting and drying (AWD) irrigation technology uptake in rice paddies of the Mekong Delta, Vietnam: relationship between local conditions and the practiced technology. Asian Afr Area Stud.

[CR81] Zhang L, Tian H, Shi H, Pan S, Qin X, Pan N, Dangal SRS (2021) Methane emissions from livestock in East Asia during 1961–2019. Ecosyst Health Sustain 7. 10.1080/20964129.2021.1918024

